# Antioxidant and Anti-Inflammatory Effects of *Chaenomeles sinensis* Leaf Extracts on LPS-Stimulated RAW 264.7 Cells

**DOI:** 10.3390/molecules21040422

**Published:** 2016-03-28

**Authors:** Young-Ki Han, Yon-Suk Kim, Sithranga Boopathy Natarajan, Won-Suk Kim, Jin-Woo Hwang, Nam-Joo Jeon, Jae-Hyun Jeong, Sang-Ho Moon, Byong-Tae Jeon, Pyo-Jam Park

**Affiliations:** 1 Korea Food Research Institute, Seongnam 13539, Korea; thwk00@naver.com; 2 Department of Biotechnology, Konkuk University, Chungju 27478, Korea; kimyonsuk@kku.ac.kr (Y.-S.K.); nsboopathy@gmail.com (S.B.N.); croucard@kku.ac.kr (J.-W.H.); hidingkking@kku.ac.kr (N.-J.J.); 3 Department of Pharmaceutical Engineering, Silla University, Busan 46958, Korea; wskim@silla.ac.kr; 4 Department of Food Science and Technology, Korea National University of Transportation, Jeungpyeong 27909, Korea; jhjeong@ut.ac.kr; 5 Korea Nokyong Research Center, Konkuk University, Chungju 27478, Korea; moon0204@kku.ac.kr (S.-H.M.); hannokwon@kku.ac.kr (B.-T.J.)

**Keywords:** anti-inflammatory, *Chaenomeles sinensis* leaf, RAW 264.7 cells, STAT1, TRIF-dependent pathways

## Abstract

The fruit of *Chaenomeles sinensis* has been traditionally used in ethnomedicine for the treatment of various human ailments, including pneumonia, bronchitis, and so on, but the pharmacological applications of the leaf part of the plant have not been studied. In this study, we evaluated the various radical scavenging activities and anti-inflammatory effects of different *Chaenomeles sinensis* leaf (CSL) extracts. The water extract showed a higher antioxidant and radical scavenging activities. However the ethanolic extracts showed higher NO scavenging activity than water extract, therefore the ethanolic extract of CSL was examined for anti-inflammatory effects on lipopolysaccharide (LPS)-stimulated RAW 264.7 cells. The 70% ethanol extract of CSL (CSLE) has higher anti-inflammatory activity and significantly inhibited the production of nitric oxide (NO), interleukin-6 (IL-6) and tumor necrosis factor-α (TNF-α). In addition, CSLE suppressed LPS-stimulated inducible nitric oxide synthase (iNOS) and NO production, IL-1β and phospho-STAT1 expression. In this study, we investigated the effect of CSLE on the production of inflammatory mediators through the inhibition of the TRIF-dependent pathways. Furthermore, we evaluated the role of CSLE on LPS-induced expression of pro-inflammatory cytokines, such as TNF-α, IL-1β and IL-6. Our results suggest that CSLE attenuates the LPS-stimulated inflammatory responses in macrophages through regulating the key inflammatory mechanisms, providing scientific support for its traditional uses in treating various inflammatory diseases.

## 1. Introduction

*Chaenomeles sinensis* (*C. sinensis*) is an important medicinal plant species of the family Rosacea. It has been traditionally used in Japan, Korea, China, Bhutan, and Burma to treat various human ailments. The medicinal properties of *C. sinensis* have been validated through the identification of bioactive components related to its pharmacological applications [1]. Specifically, β-sitosterol derived from *C. sinensis* potentially inhibited hemolysis caused by streptolysin O [2]; furthermore, the anti-inflammatory effect of an epicatechin polymer derived from *C. sinensis* was deduced from its hindrance of both the histamine release from rat mast cells and the activation of hyaluronidase [3,4]. The ethanol extracts of *C. sinensis* has also been reported for its potent antipruritic effects. The fruit of *C. sinensis* extracted by 35% ethanol successfully ameliorated chemically induced scratching behavior in mice [5]. Inflammation is a beneficial host response to a foreign challenge or tissue injury that ultimately leads to the restoration of normal tissue structure and function [6]. Inflammation is also a protective mechanism against different harmful stimuli, such as infections, tissue damage, trauma, and exposure to endotoxins like lipopolysaccharide (LPS) [7]. Macrophage activation through LPS induces production of NO, inducible NO synthase (iNOS), and pro-inflammatory cytokines such as interleukin (IL)-1β, IL-6, and tumor necrosis factor (TNF)-α, which in turn activate other macrophages and nearby cells [8,9]. However, their overproduction by activated macrophages has been implicated in the pathophysiology of many inflammatory diseases, including rheumatoid arthritis, atherosclerosis, chronic hepatitis, pulmonary fibrosis, and inflammatory brain disease [10]. LPS-stimulated macrophages are therefore used as an ideal model to study inflammation and the mechanisms of potential anti-inflammatory mediators. In this study, we aimed to evaluate the effect of *C. sinensis* leaf (CSL) extracts on the activation of macrophages and the anti-inflammatory effects by measuring cell viability, the production of pro-inflammatory cytokines (IL-6, IL-1β, and TNF-α), and the expression of the inflammatory mediators iNOS, cyclooxygenase-2 (COX-2), mitogen activated protein kinase (MAPKs) and STAT-1.

## 2. Results

### 2.1. Extraction Yields, Total Polyphenol and Flavonoid Contents of CSL Extracts

The total polyphenol content of CSL water extract and ethanol extract was 165.9 and 132.4 mg GAE/g extract, respectively. In addition, the total flavonoid content of water and ethanol extracts was 174.3 and 155 mg CE/g extract, respectively (Table 1). The content of total polyphenols and total flavonoid was higher in water extracts than in ethanol extracts.

### 2.2. Antioxidant Activity of CSL Extracts

Various antioxidant activities of CSL extracts were tested and are tabulated in Table 1. The ABTS radical scavenging activity was calculated using Trolox equivalent antioxidant capacity (TEAC) values. The TEAC values of the CSL water and ethanol extracts were recorded as 0.99 and 0.94, respectively, showing that water extract has a higher TEAC value than the ethanol extract. The FRAP assay has also been used to measure of antioxidant effects of substances. The water extract of SC has a slightly higher antioxidant effect than that of ethanol extract. However, the TEAC value and FRAP assay results indicated the strong antioxidant activities of both water and ethanol extracts. Based on the results obtained, it is highly possible that their polyphenols and flavonoids contents may contribute to the antioxidant activity of the CSL extracts [11].

### 2.3. Radical Scavenging Activity by ESR Measurement

DPPH is a stable free radical with a deep purple color. It is neutralized by reacting with other radicals, electrons, or hydrogen atoms, which is indicated by a color loss. This method has been widely used as a tool for estimating the free radical scavenging activities of antioxidants [12]. The DPPH radical scavenging activity of CSL water and ethanolic extracts closely matched the scavenging range of vitamin C (Table 2). The water extracts showed higher DPPH radical scavenging activity (IC_50_, 3.06 μg/mL), followed by ethanolic extracts (IC_50_, 5.75 μg/mL compared with vitamin C (IC_50_, 4.79 μg/mL) as positive control. These results indicate that CSL extracts have obvious scavenging effects on DPPH radical. The alkyl radical spin adduct of 4-POBN/free radical was generated from AAPH. The water extracts showed higher alkyl radical scavenging activity followed by ethanolic extract and it was compared to that of vitamin C (Table 2). Alkyl radical scavenging activity of water and ethanol extract of CSL was (IC_50_, 0.85 μg/mL), (IC_50_, 7.00 μg/mL) respectively and they were compared with vitamin C (IC_50_, 5.70 μg/mL) as positive control. The hydroxyl radical scavenging activities (IC_50_ values) of water and ethanol extract of CSL and the positive control vitamin C were 205.45, 328.58 and 42.41 μg/mL, respectively. The water extract of CSL showed a lower hydroxyl radical scavenging activity than vitamin C, however, it was higher than that of ethanol extract. Taken together these results indicate that CSL extracts showed strong DPPH, alkyl and hydroxyl radical scavenging activities and suggest that CSL has potential antioxidant and anti-inflammatory effects.

### 2.4. Cell Viability and Production of NO

**Figure 1:**
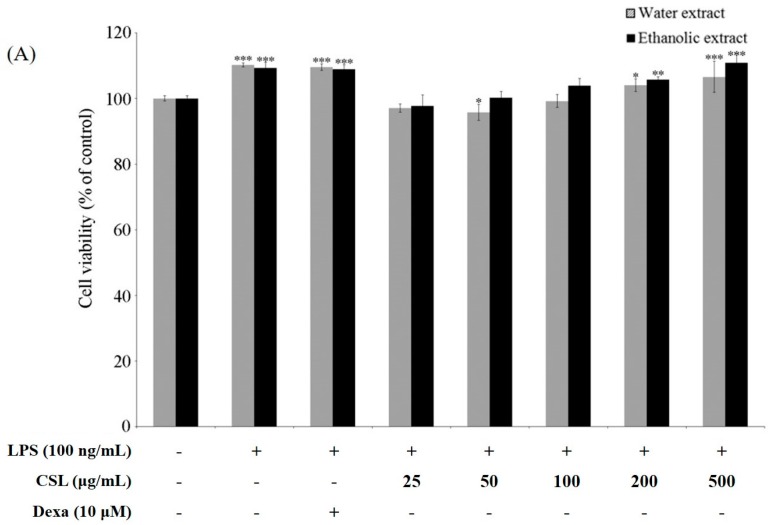

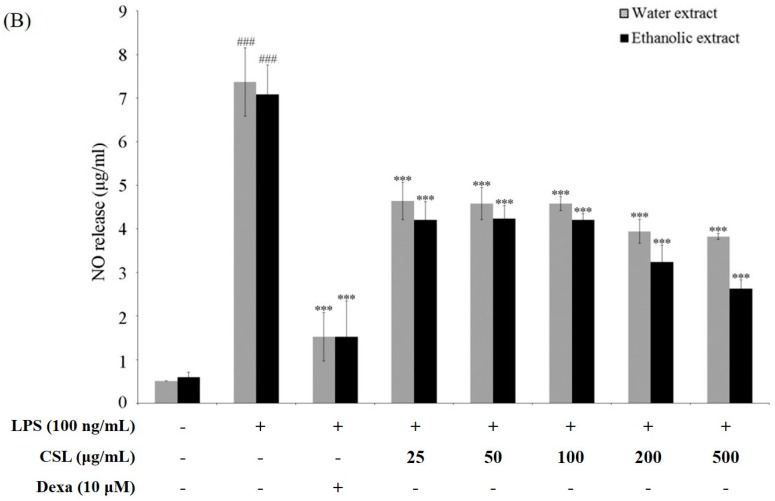
Cell viability and inhibition of nitrite production (**A**) RAW 264.7 cells were pretreated with extracts of CSL for 1 h (at 25, 50, 100, 200, 500 µg/mL), and then treated with LPS (100 ng/mL) for 18 h. Cell viability was measured with the MTT assay. Data are represented as mean ± SD (*n* = 4) with a one-way ANOVA followed by the Dunnett’s test. * *p* < 0.05, ** *p* < 0.01 and *** *p* < 0.001 *vs.* control; (**B**) RAW 264.7 cells were pretreated for 1 h with extracts of CSL and then treated with LPS (100 ng/mL) for 18 h. The concentration of NO in the culture medium was determined by using the Griess assay. Data are represented as mean ± SD (*n* = 4) with a one-way ANOVA followed by the Dunnett’s test. ***^###^*** *p* < 0.001 *vs.* control, *** *p* < 0.001 *vs.* LPS.

RAW 264.7 cells were pretreated with extracts of CSL (25, 50, 100, 200, and 500 µg/mL) for 1 h prior to stimulation with LPS (100 ng/mL) for 18 h. The results of the MTT assay indicated that CSL was not cytotoxic to RAW 264.7 cells at concentrations up to 500 mg/mL (Figure 1A). As shown in Figure 1B, the cells that were treated with LPS alone showed a marked increase in NO release as compared with the non-treated cells, while CSL inhibited the levels of NO in a dose-dependent manner. Also, the ethanol extracts showed higher inhibition of the NO levels compared to that of the water extracts. The water extracts showed higher effect than ethanol extract on various radical scavenging activity. However ethanol extract was potent in inhibiting the NO as compared to water extract in RAW 264.7 cell. We therefore selected 70% ethanol extracts of CSL (CSLE) with concentrations of 50, 100, and 200 µg/mL for further study.

### 2.5. Inhibition of LPS-Induced Proinflammatory Cytokine Production by CSLE in LPS-Stimulated RAW 264.7 Cells

**Figure 2:**
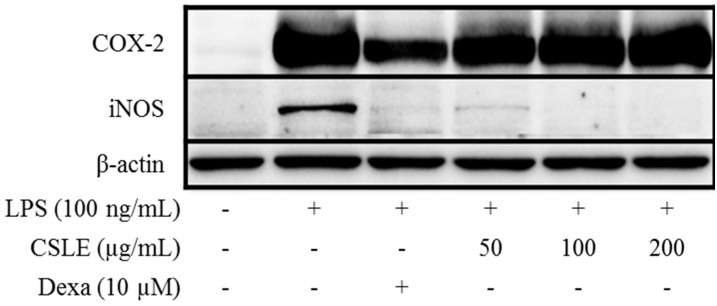
iNOS and COX-2 protein expression in LPS-stimulated RAW 264.7 macrophage. Cells were pretreated with CSLE (50, 100, and 200 μg/mL) and dexamethasone (10 µM) for 1 h before exposure to LPS (100 ng/mL) for 3 h (COX-2) and 18 h (iNOS). Cytosolic lysates were separated using SDS-PAGE. The iNOS, COX-2, and β-actin were detected using western blot analysis.

**Figure 3:**
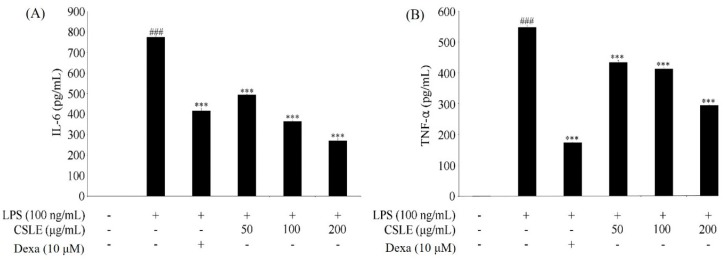
Pro-inflammatory cytokine inhibitory effects of CSLE in LPS-stimulated RAW 264.7 macrophages. Cells were pretreated with CSLE (50, 100, and 200 μg/mL) for 1 h before exposure to LPS (100 ng/mL) for 18 h (**A**) IL-6, and 3h (**B**) TNF-α. The IL-6 and TNF-α were detected using ELISA. Results are expressed as mean ± SD from three independent experiments. ^###^ *p* < 0.001 compared to control, and *** *p* < 0.001 compared to LPS alone.

**Figure 4:**
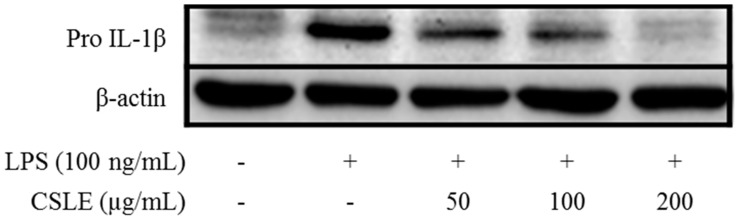
Pro IL-1β protein expression in LPS-stimulated RAW 264.7 macrophage. Cells were pretreated with CSLE (50, 100, and 200 μg/mL) for 1 h before exposure to LPS (100 ng/mL) for 4 h (pro IL-1β). Cytosolic lysates were detected using western blot analysis.

It is well known that NO is produced by iNOS in the cells. To determine if the effect of CSL on NO production was due to a decrease in iNOS expression, the levels of iNOS were quantitated using an immunoblot assay. As shown in Figure 2, stimulation of the RAW 264.7 cells with LPS (100 ng/mL) increased the level of iNOS. However, CSLE remarkably inhibited the protein expression level of iNOS. To determine the potential effects of CSLE on the production of pro-inflammatory cytokines, we examined the accumulation of TNF-α, IL-6 and IL-1β. CSLE inhibited the protein levels of TNF-α, IL-6 and pro-IL-1β (Figure 3 and Figure 4). These data suggest that CSLE has the ability to downregulate LPS-induced NO, TNF-α, IL-6 and pro-IL-1β production.

### 2.6. Inhibitory Effects of CSLE on ROS Generation

We also investigated the effect of CSLE on LPS-induced intracellular reactive oxygen species (ROS) production using the fluorescent probe DCFH-DA, which can be oxidized to the highly fluorescent compound DCF. CSLE effectively suppressed LPS-induced intracellular ROS generation as shown by NAC.

**Figure 5:**
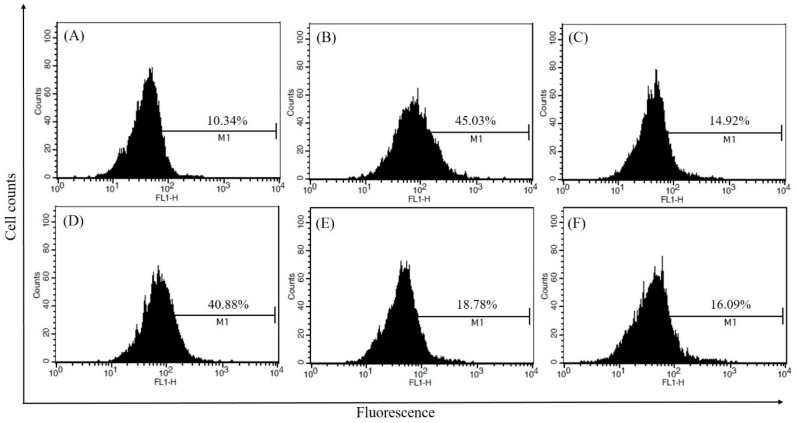
Intracellular ROS determination using DCF-DA on the LPS-stimulated RAW264.7 macrophages. (**A**) Control; (**B**) LPS; (**C**) LPS + NAC (20 mM); (**D**) LPS + CSLE (50 μg/mL); (**E**) LPS + CSLE (100 μg/mL) and (**F**) LPS + CSLE (200 μg/mL).

This finding may clarify the anti-inflammatory activity of CSLE, which presumably works by removing the free radicals that participate in the activation of the inflammatory response, thereby contributing to cellular damage. As shown in Figure 5, the LPS-induced intracellular ROS production was increased by approximately 35% compared to the control. Due to the treatment with CSLE, the ROS production was decreased in a dose-dependent manner. These results indicate that CSLE can regulate intracellular ROS.

### 2.7. Absence of CSLE Affect on MyD-88 Dependent Signaling Pathways

**Figure 6:**
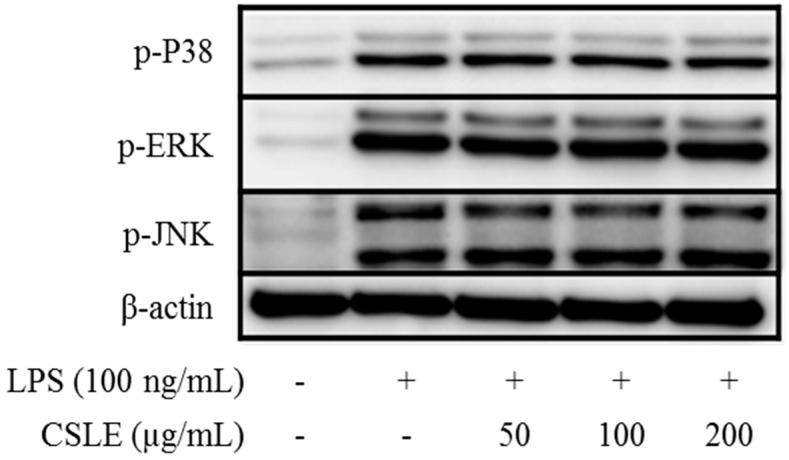
MyD88-dependent pathway associated protein expression in LPS-stimulated RAW 264.7 macrophages. Cells were pretreated with CSLE (0.05, 0.1, and 0.2 mg/mL) for 1 h before exposure to LPS (100 ng/mL) for 15 min. MAPK (p38, ERK1/2, and JNK) phosphorylation was detected using a western blot analysis.

To understand the mechanism underlying the inhibition of the LPS-induced production of NO and pro-inflammatory cytokines, we examined the effects of CSLE on the LPS-induced Toll-like receptor (TLR)4 signaling pathway in the RAW 264.7 cells. LPS is recognized by TLR4 and leads to the activation of the following two different signaling pathways: the MyD88-dependent and TRIF-dependent pathways. The MyD88-dependent pathway plays a critical role in the regulation of macrophage activation, as MyD88 activates the mitogen activated protein kinases (MAPKs). It is known that the LPS-induced phosphorylation of MAPKs and the inhibitor of kappa B (IκB)-α lead to the expression of proinflammatory mediators in macrophages [13]. To investigate the effect of CSLE on the MAPKs pathway, we examined the phosphorylation of three MAPKs. As shown in Figure 6, CSEL did not inhibit the LPS-induced phosphorylation of JNK, ERK and p38; also, the CSLE did not inhibit LPS-induced degradation of IκB-α. These data indicate that CSLE does not regulate the LPS-induced MyD88 dependent signaling pathway.

### 2.8. TRIF-Dependent Signaling Pathways Inhibit CSLE

**Figure 7:**
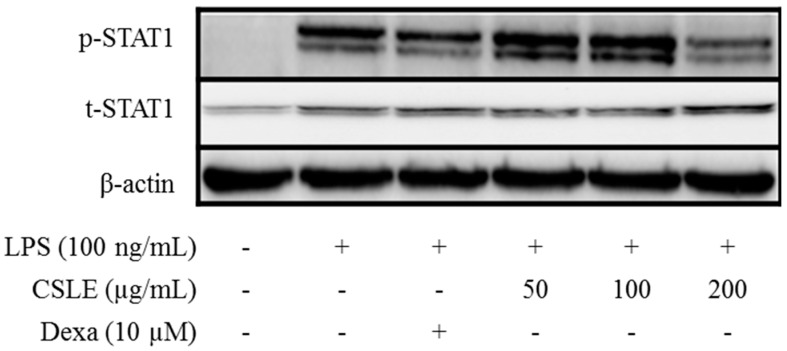
Effect of p-STAT1 expression by extract in LPS-stimulated RAW264.7 macrophages. Cells were pretreated with CSLE (0.05, 0.1, and 0.2 mg/mL) and dexamethasone (10 µM) for 1 h before exposure to LPS (100 ng/mL) for 3 h. Cytosolic lysates were separated using SDS–PAGE. Total-STAT1, phospho-STAT1 and β-actin were detected using a western blot analysis.

**Figure 8:**
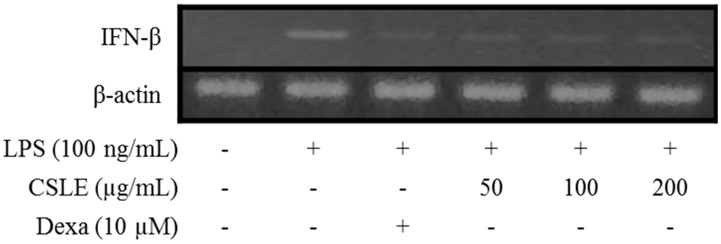
The effects of CSL on LPS-induced rise of IFN-β mRNA levels in RAW264.7 cells. Cells were pretreated with CSLE (0.05, 0.1, and 0.2 mg/mL) for 1 h before exposure to LPS (100 ng/mL) for 3 h. Total RNA was isolated, IFN-β mRNA were determined using RT-PCR, and β-actin mRNA was used as control.

The TRIF-dependent signals after LPS stimulation lead to the activation of the interferon regulatory factor (IRF)-3 and interferon (IFN)-β expression [14]. Secreted IFN-β is recognized by the receptor for Type I IFN and triggers the activation of JAK/STAT1 signaling which mediates the expression of inducible genes such as iNOS [15]. STAT signaling is critical for IL-6 and IL-1β production [16]. Therefore, we investigated the inhibitory effect of CSLE on STAT1 phosphorylation using an immunoblot analysis. As shown in Figure 7, CSLE (200 μg/mL) attenuated LPS-induced STAT1 phosphorylation and inhibited IFN-β production (Figure 8). These results indicate that CSLE inhibited STAT1 phosphorylation via the attenuation of IFN-β production.

## 3. Discussion

The higher antioxidant and radical scavenging activities of water extracts indicated the presence of active water soluble compound in the leaves and also higher amount of polyphenolic and flavonoid contents present in the CSL extracts (Table 1). Plant-derived polyphenols and flavonoids are major groups of metabolites which play an important role in the field of biomedicine. The antioxidant and radical scavenging activity of polyphenols and flavonoids are mainly responsible for the redox properties of the extract which can play an important role in neutralizing the free radicals [17]. In the present study, the water extracts showed higher antioxidant activity than the ethanolic extract. However, the ethanolic extract showed higher NO scavenging activity than water extracts. These results indicate the multifunctional nature of the diverse group of compounds present in CSL extract. Therefore the ethanolic extract was chosen to study its anti-inflammatory effects in LPS stimulated RAW cells.

LPS is well known to activate two following downstream pathways: the MyD88 and TRIF-dependent pathways. LPS-induces nuclear factor kappa B (NF-κB) and MAPK are activated by the MyD88-dependent pathway, which leads to increased productions of inflammatory cytokines [13]. In the MyD88 pathway, the phosphorylation of MAPKs acts as a transcriptional activator of AP-1 expression. Additionally, COX-2 expression is mediated via LPS-induced NF-κB activation [18,19]; these studies have demonstrated that the activation of NF-κB is required for LPS-induced COX-2 expression. The TRIF-dependent signal induces the expression of IFN-β [20]. In this pathway, the phosphorylation of IRF-3 acts as a transcriptional activator of IFN-β expression and leads to STAT1 phosphorylation, which acts as a crucial transcriptional factor for iNOS gene in LPS-stimulated mouse macrophages [14]. Numerous studies have demonstrated that natural compounds suppress the inflammatory response by inhibiting the production of pro-inflammatory mediators [21,22]. Several studies demonstrated that natural derivatives decrease the LPS-induced NO production in RAW 264.7 macrophage cells through the inhibition of iNOS expression [23,24]. Accordingly, our study shows analogous results, wherein the CSLE inhibited the increase of the NO and iNOS expression levels in LPS-stimulated RAW 264.7 cells. These results demonstrate that the CSLE decreased the NO production by inhibition of iNOS expressions levels. Also, the CLSE did not have inhibitory effects on the phosphorylation levels of the MAPKs (ERK, JNK and p38). Furthermore, the CSLE did not affect the IκB-α degradation and COX-2 expression, but it did significantly inhibit the expression of iNOS. Our results therefore indicate that the inhibitory effect of CSLE on NO production and iNOS expression is mediated by suppression of the TRIF-dependent signaling pathway. LPS-stimulated RAW 264.7 cells can induce pro-inflammatory cytokines under inflammatory conditions. Several studies have reported that pro-inflammatory cytokines such as TNF-α, IL-6, and IL-1β induce the expression of iNOS and NO production by activating NF-κB and MAPK [25,26]. We showed that CSLE significantly inhibited the production of TNF-α, IL-6 and IL-1β in LPS-stimulated RAW 264.7 cells, indicating that CSLE could suppress the inflammatory response through the inhibition of inflammatory cytokines. The TRIF-dependent pathway response of LPS-stimulated RAW 264.7 cells plays an important role in type 1 IFN induction, particularly regarding IFN-β. The TRIF interacts with TANK-binding kinase (TBK) 1, which mediates IRF3 activation, and then leads to the increase of IFN-β [13]. IFN-β plays a critical role in the expression of iNOS via the phosphorylation of STAT1 in immune responses [27]. Therefore, we investigated the role of CSLE on IFN-β/STAT1 pathway that is known to be activated by LPS-stimulation in RAW 264.7 cells. We found that the CSLE inhibited LPS-induced IFN-β production and STAT1 phosphorylation in the RAW 264.7 cells. Our results show that CSLE inhibits NO production and iNOS expression by the suppression of the IFN-β/STAT1 pathway in LPS-stimulated RAW 264.7 cells. Therefore, our results suggest that CSLE may modulate the TRIF-dependent pathway to elicit its anti-inflammatory effects.

## 4. Materials and Methods

### 4.1. Chemicals and Reagents

Dulbecco’s modified eagle’s medium (DMEM), fetal bovine serum (FBS), penicillin and streptomycin were purchased from Hyclone (Thermo Scientific, Waltham, MA, USA). Antibodies for phosphorylated-ERK, -p38, -JNK, iNOS and COX-2 were purchased from Santa Cruz Biotechnology Inc. (Dallas, TX, USA). Antibodies for phosphorylated-IκBα, -STAT1 and β-actin, were purchased from Cell Signaling Technology Inc. (Denvers, MA, USA). Mouse IL-6 enzyme-linked immunosorbent assay (ELISA) kits and Mouse TNF-α ELISA kits were purchased from BD Biosciences (San Diego, CA, USA). The detection agents and polyvinylidine fluoride (PVDF) membrane were purchased from GE Healthcare Life Sciences (Little Chalfont, Buckinghamshire, UK). LPS, 2′,7-dichlorofluorescein diacetate (DCFH-DA) and 3-(4,5-dimethylthiazol-2-yl)-2,5-diphenyltetrazolium bromide (MTT) were purchased from Sigma Chemical Co. (St. Louis, MO, USA). All of the other reagents were of the highest commercially available grade.

### 4.2. Preparation of the Extract

The leaves of *C. sinensis* were purchased in Chungju in the summer of 2010. The *C. sinensis* leaf (CSL) was washed and lyophilized for the experiment. The CSL was extracted in water and 70% ethanol. The water extract was prepared by hot water extraction of dried CSL (50 g) for 2 h in 1 L of water at 90 °C. The ethanol extracts was prepared twice with 70% ethanol for 1 day and then filtered with a Whatman No. 41 filter at room temperature (R.T.). The filtrate was evaporated using an evaporator (EYELA, Tokyo, Japan) at 50 °C. After evaporation, the water and ethanol extracts were lyophilized and stored at −20 °C until use.

### 4.3. Antioxidant Activity of the Chaenomeles Sinensis Leaves Extract

#### 4.3.1. Determination of Total Polypenol Contents

Total phenolic contents of CSL extracts were determined using the Folin-Ciocalteu assay [28]. Briefly, 10 mg of CSL extracts were dissolved individually in 10 mL of D.W, to which 0.1 mL of reaction mixture containing 50 μL of 50% Folin-Ciocalteau reagent, and 150 μL of 20% sodium carbonate (Na_2_CO_3_) was added. After incubation at R.T. for 30 min, the absorbance of the reaction mixtures was measured at 760 nm by a spectrophotometer (SECOMAM, Ales, France). Gallic acid was used as a standard, and the total polyphenol contents of CSL extracts were expressed in milligram gallic acid equivalent (mg GAE/g extract).

#### 4.3.2. Determination of Total Flavonoid Contents

Total flavonoid contents were determined by the aluminum colorimetric method and the catechin was used as standard [29]. Briefly, the test samples were individually dissolved in D.W. (150 μL), and added to a reaction mixture containing 150 μL of 2% AlCl_3_. After 10 min of incubation at ambient temperature, the absorbance of the supernatant was measured at 510 nm by using a spectrophotometer. The total flavonoid content was expressed as catechin equivalents in milligram per gram extract (mg CE/g extract).

#### 4.3.3. ABTS Radical Scavenging Activity

The total antioxidant activity of the CSL extracts was measured by the ABTS radical cation de-colorization assay involving the preformed ABTS radical cation [30]. ABTS radical cation (ABTS^•+^) was produced by the 7 mM ABTS stock solution with 2.45 mM potassium persulfate (K_2_S_2_O_8_) and allowing the mixture to stand in the dark at R.T. for 14 h before use. To determine the scavenging activity, 0.9 mL of ABTS reagent was mixed with 0.1 mL of extracts and the absorbance was measured at 734 nm after 6 min of reaction at R.T. The total antioxidant activity of the CSL extracts was expressed by Trolox equivalent antioxidant capacity (TEAC) values.

#### 4.3.4. Ferric Reducing Antioxidant Power (FRAP) Assay

The FRAP was assayed by the method of Benzie and Strain [31]. An aliquot of a FRAP reagent (3 mL) containing a mixture of 0.3 M acetate buffer, 10 mM TPTZ in 40 mM HCl, and 20 mM ferric chloride (10:1:1 *v*/*v*/*v*), were combined with 1 mL of sample. The absorbance values were compared with those obtained from the standard curves of FeSO_4_ (0–10 mM). The antioxidant capacity of individual sample was expressed as mM FeSO_4_ equivalent in mg extract (mM FeSO_4_ eq./mg extract).

#### 4.3.5. Radical Scavenging Activity by ESR Measurement

##### DPPH Radical Scavenging Activity

The DPPH radical scavenging activity was measured using an ESR spectrometer (JES-FA machine; Jeol, Tokyo, Japan) according to the method described by Kim *et al.* [32]. Thirty microliters of each sample was added to 30 μL of DPPH (60 μM) dissolved in ethanol and ethanol alone was used as a control. After 10 s of vigorous mixing, the solutions were transferred to Teflon capillary tubes and fitted into the cavity of the ESR spectrometer. The spin adducts were determined by the ESR spectrometer 2 min later under the measurement conditions.

##### Alkyl Radical Scavenging Activity

The alkyl radical scavenging activity was assayed by the method of Hiramoto *et al.* [33]. The alkyl radicals were generated by 2,2-azobis(2-amidinopropane) hydrochloride (AAPH). The sample (20 μL) was added to a reaction mixture containing 20 μL of PBS (pH 7.4), 40 mM AAPH and 40 mM 4-POBN. After incubation at 37 °C in water bath for 30 min, the samples transferred to a Teflon capillary tube and the spin adduct was measured.

##### Hydroxyl (OH) Radical Scavenging Activity

The OH radical scavenging activity was measured by the method of Rosen *et al.* [34]. Hydroxyl radicals were generated through the Fenton reaction, and reacted rapidly with the nitrone spin trap DMPO. The resultant DMPO-OH adducts were detected using an ESR spectrometer. Samples (20 μL) were added to reaction mixtures containing 20 μL of 0.3 M DMPO, 20 μL of 10 mM FeSO_4_, 20 μL of 10 mM H_2_O_2_, reacted for 2.5 min and then transferred to a Teflon capillary tube for the determination of spin adduct formation.

### 4.4. Cell Culture

The murine macrophage cell line RAW 264.7 was obtained from the American Type Culture Collection (ATCC, TIB-71™), and was maintained in Dulbecco’s modified Eagle’s medium (DMEM) medium supplemented with 10% heat-inactivated fetal bovine serum (FBS) and antibiotics (100 units/mL of penicillin and 100 μg/mL of streptomycin) at 37 °C in a humidified incubator containing 5% CO_2_.

### 4.5. Determination of Cell Viability Assay

The cell viability effect of CSL extract was evaluated using the MTT colorimetric assay. The RAW 264.7 cells were plated at a density of 1 × 10^4^ cells/well into 96-well plates at 18 h before treatment. The cells were treated with various concentrations (25, 50, 100, 200, and 500 μg/mL) of the CSL and dexamethasone (positive control) and they were stimulated with or without medium containing LPS (100 ng/mL) at 37 °C for 24 h. After treatment, the medium was replaced by 100 μL of the DMEM medium containing MTT (500 µg/mL) in each well and followed by incubation at 37 °C for 4 h. The MTT solution was then discarded and the intracellular formazan product was dissolved in 200 μL DMSO with shaken for 5 min. The absorbance was measured at 540 nm using a microplate reader (Tecan, Grödig, Austria) and values were calculated in comparison to the control cells.

### 4.6. Determination of Nitric Oxide (NO) Production

The nitrite oxide concentration in the culture medium was determined using the Griess reagent. The RAW 264.7 cells were plated at a density of 1 × 10^4^ cells/well into 96-well plates and pre-treated with various concentrations (25, 50, 100, 200, and 500 μg/mL) of CSL and dexamethasone. The cells were stimulated with medium containing LPS (100 ng/mL) at 37 °C for 24 h. The culture supernatant (100 µL) was mixed with Griess reagent (100 µL, 1% sulfanilamide, 0.1% *N*-1-naphthylethylenediamine) and incubated at room temperature for 10 min. The absorbance of the mixture was determined at 540 nm using a microplate reader. A range of dilutions of sodium nitrite was used for a standard curve with the amount of nitrite in each sample.

### 4.7. Determination of Intracellular ROS Using Flow Cytometry

Intracellular ROS levels were measured by detecting the fluorescent intensity of cells as described by Bass *et al.* [35]. RAW 264.7 cells were plated at a density of 1 × 10^6^ cells/well into 6-well plates, pre-treated with various concentrations (50, 100, and 200 μg/mL) of CSLE and *N*-acetyl-l-cysteine (NAC, 20 mM) for 1 h. The cells were stimulated with of medium containing LPS (100 ng/mL) at 37 °C for 30 min. Cells were washed with PBS and incubated at 37 °C for 30 min (in dark) with the probe at a final concentration of 10 µM DCF-DA. Cells were washed with PBS and gently scraped. The fluorescent intensity was analyzed at an excitation wavelength of 485 nm and an emission wavelength of 535 nm using a FACS Calibur flow cytometer (Becton & Dickinson Co., Franklin Lakes, NJ, USA).

### 4.8. Measurement of CYtokine (IL-6 and TNF-α)

The RAW 264.7 cells were plated at a density of 4 × 10^4^ cells/well in 24-well plates, pre-treated with various concentrations (50, 100, and 200 μg/mL) of CSLE and dexamethasone (10 μM) for 1 h. the cells were stimulated with medium containing LPS (100 ng/mL) at 37 °C for 3 h and 18 h. The levels of TNF-α and IL-6 in cultured medium (supernatant) were measured with enzyme-linked immunosorbent assay (ELISA) kit (BD OptEIA) in accordance with the manufacturer’s protocols. The absorbance was measured at 540 nm using a microplate reader.

### 4.9. Western Blot Analysis

The RAW 264.7 cells were plated at a density of 2 × 10^6^ cells in a 6-well plate, pre-treated with various concentrations (50, 100 and 200 μg/L) of CSLE and dexamethasone (10 μM) for 1 h. The cells were stimulated with the medium containing LPS (100 ng/mL) at 37 °C for 24 h. The cells were subsequently washed with PBS, collected and suspended in a lysis buffer (150 mM NaCl, 10 mM Tris (pH 7.5), 5 mM EDTA, and 1% Triton X-100) containing protease inhibitors (1 μg/mL leupeptin and 100 μg/mL PMSF), and centrifuged at 12,000 g in 4 °C for 20 min to yield cell lysates. The protein level in each sample was measured using a protein assay kit (Bio-Rad, Laboratories, Inc., Hercules, CA, USA). The proteins (20 μg of RAW 264.7 lysates) were separated with 10% SDS polyacrylamide gels and transferred to polyvinylidene difluoride (PVDF) membranes (GE Healthcare, Buckinghamshire, UK). The membranes were then blocked by Tris-buffered saline-Tween 20 solution (TBS-T) containing 5% non-fat dry milk, and incubated sequentially with primary antibody and horseradish peroxidase-conjugated anti-mouse or anti-rabbit IgG (Bio-Rad). The protein bands were then visualized using an ECL detection kit and a luminescent image analyzer (LAS-3000, Fujifilm, Tokyo, Japan).

### 4.10. Reverse Transcription-Polymerase Chain Reaction (RT-PCR)

The RAW 264.7 cells were plated at a density of 2 × 10^6^ cells in a 6-well plate, pre-treated with various concentrations (50, 100, and 200 μg/mL) of CSLE and dexamethasone (10 μM) for 1 h. The cells were stimulated with the medium containing LPS (100 ng/mL) at 37 °C. After a 2 h incubation, the total RNA was isolated from cells using TRIzol reagent according to the manufacturer’s instructions. To make cDNA, 2 μg of RNA was reversely transcribed using Oligo (dT) 18 primer and AccuPower™ RT Premix.

### 4.11. Statistical Analysis

The data are expressed as the mean standard deviation for triplicate determinations. Analysis of variance (ANOVA), together with Tukey’s test and Dunnett’s test (GraphPad Prism 5), was conducted to identify the significant differences between the samples (*p* < 0.05).

## 5. Conclusions

Our results demonstrate that CSLE inhibits the production of NO, iNOS expression, and pro-inflammatory cytokines (TNF-α, IL-6, and IL-1β) in LPS-stimulated RAW 264.7 macrophages. Additionally, the CSLE inhibited the phosphorylation of STAT1 and the production of IFN-β in LPS-stimulated RAW 264.7 macrophages. Therefore, we suggest that CSLE should be considered as potential anti-inflammatory candidate for the treatment of inflammatory diseases. Further studies are required to elucidate the specific anti-inflammatory active compound of CSLE, as well as its clinical therapeutic potential.

## Figures and Tables

**Table 1:** Total polyphenol and flavonoid content of CSL extract.

Sample	Extraction Yield (%)	Total Polyphenol (mg GAE/g Extract)	Total Flavonoid (mg CE/g Extract)	ABTS (mM Trolox eq./mg Extract)	FRAP (mM FeSO_4_ eq./mg Extract)
Water extract	28.36	165.9 ± 2.5	174.3 ± 3.2	0.99 ± 0.04	2.45 ± 0.08
Ethanol extract	26.7	132.4 ± 1.9	155 ± 4.2	0.94 ± 0.03	2.16 ± 0.07

**Table 2:** Various radical scavenging activities of CSL extract (IC_50_, μg/mL).

Sample	DPPH Radical	Alkyl Radical	Hydroxyl Radical
Water extract	3.06 ± 0.12	0.85 ± 0.02	205.45 ± 5.98
Ethanol extract	5.75 ± 0.24	7.00 ± 0.36	328.58 ± 6.47
Vitamin C	4.79 ± 0.25	5.70 ± 0.32	42.41 ± 2.31
